# Harnessing the Therapeutic Potential of Decoys in Non-Atherosclerotic Cardiovascular Diseases: State of the Art

**DOI:** 10.3390/jcdd8090103

**Published:** 2021-08-27

**Authors:** Maryam Mahjoubin-Tehran, Stephen L. Atkin, Evgeny E. Bezsonov, Tannaz Jamialahmadi, Amirhossein Sahebkar

**Affiliations:** 1 Student Research Committee, Mashhad University of Medical Sciences, Mashhad, Iran; mahjoubintm951@mums.ac.ir; 2 Department of Medical Biotechnology and Nanotechnology, Faculty of Medicine, Mashhad University of Medical Sciences, Mashhad, Iran; 3 Weill Cornell Medicine Qatar, Doha, Qatar; stlatkin@gmail.com; 4 Laboratory of Angiopathology, Institute of General Pathology and Pathophysiology, 8 Baltiiskaya Street, 125315 Moscow, Russia; evgeny.bezsonov@gmail.com; 5 Laboratory of Cellular and Molecular Pathology of Cardiovascular System, Institute of Human Morphology, Moscow, Russia; 6 Department of Biology and General Genetics, I.M. Sechenov First Moscow State Medical University (Sechenov University), Moscow, Russia; 7 Department of Food Science and Technology, Quchan Branch, Islamic Azad University, Quchan, Iran; jamiat931@gmail.com; 8 Department of Nutrition, Faculty of Medicine, Mashhad University of Medical Sciences, Mashhad, Iran; 9 Biotechnology Research Center, Pharmaceutical Technology Institute, Mashhad University of Medical Sciences, Mashhad, Iran; 10 Applied Biomedical Research Center, Mashhad University of Medical Sciences, Mashhad, Iran; 11 School of Medicine, The University of Western Australia, Perth, Australia; 12 School of Pharmacy, Mashhad University of Medical Sciences, Mashhad 9177948954, Iran

**Keywords:** cardiovascular diseases, decoy, oligodeoxynucleotide, peptide

## Abstract

Cardiovascular disease (CVD) is the main cause of global death, highlighting the fact that conventional therapeutic approaches for the treatment of CVD patients are insufficient, and there is a need to develop new therapeutic approaches. In recent years, decoy technology, decoy oligodeoxynucleotides (ODN), and decoy peptides show promising results for the future treatment of CVDs. Decoy ODN inhibits transcription by binding to the transcriptional factor, while decoy peptide neutralizes receptors by binding to the ligands. This review focused on studies that have investigated the effects of decoy ODN and decoy peptides on non-atherosclerotic CVD.

## 1. Introduction

Cardiovascular diseases (CVDs) are the main cause of global mortality, in spite of many years of research and development [1], resulting in an increasing healthcare burden globally [2,3]. Moreover, established CVD increases the severity of other diseases such as coronavirus disease 2019 (COVID-19), which still continues as a global pandemic [4], and it has been shown that CVD is a risk factor for COVID-19 mortality [5].

**Figure 1:**
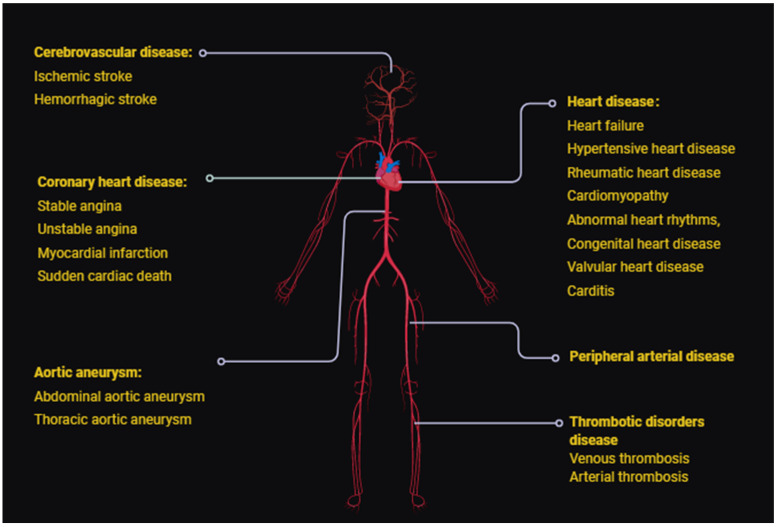
A schematic illustration of common types of CVDs including cerebrovascular disease, coronary heart disease, aortic aneurism, heart disease, peripheral artery disease, and thrombotic Disorders.

CVD encompasses a wide variety of diseases including cerebrovascular disease, coronary artery disease, aortic aneurysm, heart disorders, peripheral arterial disease, and thromboembolic disease, often resulting in compromised blood flow to the target organ. (Figure 1). Hypertension, high blood cholesterol, an unhealthy diet, drugs, physical inactivity, infection, and inflammation are common risk factors of CVD ([6]). The limitations of conventional therapies have led to the investigation of new therapeutic approaches. In recent years, the potential clinical utility of nucleic acid-based therapeutics such as small interfering RNAs (siRNAs), antisense oligonucleotides (ASOs), microRNAs, and decoy oligodeoxynucleotides (ODNs) have attracted attention. The efficacy of nucleic acid-based pharmacology has been demonstrated in clinical trials [7,8].

## 2. Decoys

A decoy is defined as something that is aimed to draw attention away from a particular situation or from an intended course of action. This is the premise of decoy base therapy where the drug candidates represent an opportunity to inhibit a known activated regulatory pathway that promotes CVD. Decoy technology includes two main approaches, decoy ODN and decoy peptides [9,10].

**Figure 2:**
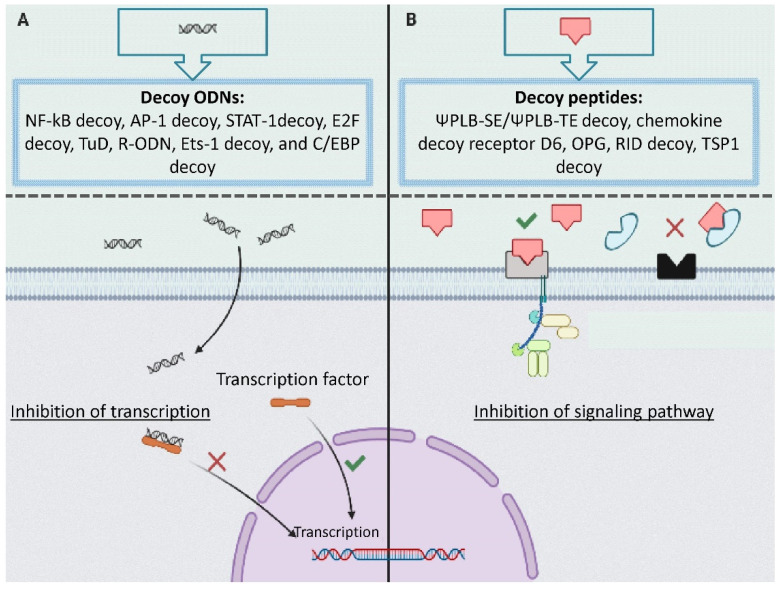
The mechanism of action of decoys: (**A**) decoy oligodeoxynucleotides (ODN) including NF-kB decoy, AP-1 decoy, STAT-1decoy, E2F decoy, tough decoy, R-ODN, and C/EBP decoy inhibits transcription by binding to the transcriptional factor; (**B**) decoy peptide including ΨPLB-SE/ΨPLB-TE decoy, chemokine decoy receptor D, decoy receptor osteoprotegerin neutralizes receptors by binding to the ligands.

Decoy ODN is a DNA construct that regulates the expression of genes related to disease through a reduction of authentic *cis–trans* interaction. Transcription factors, as *trans*-regulatory elements, regulate gene transcription by binding to the *cis*-element [11]. Decoy ODNs have a similar structure to the *cis*-element of the target gene and trap the transcription factor and modulate the gene expression (Figure 2A) [9].

Many pathological processes related to CVD such as inflammation are triggered by associated ligand–receptor interactions [12]. Peptide decoys, also known as decoy receptors, are proteins that have a similar structure to receptors. Peptide decoys can bind to specific ligands and trap them resulting in attenuated ligand–receptor interactions (Figure 2B).

### Delivery of Decoy

The efficacy of decoys mostly relies on their stability and delivery. Local delivery is considered the most effective approach for overcoming systemic administration problems; however, local delivery is only possible for a few organs such as the lungs and the eyes [13]. Generally, in systemic delivery, most mammalian cells could take up sufficient decoys; however, to maximize cellular uptake and increasing efficiency under physiological conditions, active transport delivery systems and/or receptor-mediated endocytosis should be considered [13]. Moreover, decoy delivery can also be regarded as passive (for tumor and other highly permeable organs) or active targeting delivery (for peripheral tissues) [14].

Various delivery systems for decoys may be employed, including electrically enhanced transfer, pressure-mediated transfer, biolistic bombardment, lipidoids, cationic liposomes, lipid-based nanoparticles, virus of Japan (HVJ) liposomes (the delivery time is approximately 15–30 min and sustainability up to 1–2 weeks), bacterial vectors, hemagglutinating microsphere-aided delivery, steroid mediated gene transfer, peptide-mediated delivery, and aptamer/oligonucleotide chimeras, exosomes and cell-based carriers [13]. For in vivo delivery, liposomes and viral vectors such as adenoviruses, adeno-associated virus (AAV), and lentivirus are mostly used in mammalian cells [14]. The non-viral delivery systems can be further divided into polymer, lipid, protein, and peptide-based delivery methods [13]. Poly(D, L-lactide co-glycolide) and chitosan), gelatin, liposomes, and lipid-based nanoparticles, palmitoyl-oleyl-phosphatidylcholine (POPC), poly(glycoamidoamine)s, and biodegradable polymer d,l lactide-co-glycolide (PLGA) are some examples of carriers for decoy delivery [13].

## 3. Therapeutic Potential of Decoys

The application of decoy technology as a therapeutic tool has been considered in various diseases. The ACE2 receptor is critical for viral SARS-CoV-2 entry. Recombinant human ACE2 peptide decoy performing as a soluble molecular trap for SARS-CoV-2 prevents attachment of viral particles to ACE2 receptors on the cell surface; therefore, it may have utility in treating COVID-19 disease [13]. ACE2 decoy utility as a therapy for COVID-19 disease is underway in clinical trials (NCT04324996). Furthermore, the FDA-approved anticoagulation agent heparin may act as a decoy and effectively neutralize SARS-CoV-2 by binding to the surface spike protein of the virus, which is essential for the attachment onto human cells and for the infection onset [14].

Additionally, the application of decoys for the treatment of cancer, another major global health problem, has been investigated. Reports for the usage of decoy ODN for reducing the expression of target genes including ER, CRE, STAT3, and Sp1 have shown potential therapeutic utility in breast cancer therapy [9]. It has also been shown that peptide decoys have a potential therapeutic effect for cancers such as breast cancer [10], bladder cancer [15], colon cancer [16], lung cancer [17], liver cancer [18], and prostate cancer [19]. In this review, we summarized studies that have investigated the effects of ODN and peptide decoys on non-atherosclerotic CVD (Table 1).

## 4. Effects of Decoys on Non-Atherosclerotic CVD

### 4.1. Nuclear Factor-Kappa B

The nuclear factor-B (NF-κB) family mediates the processes of inflammation, cell differentiation, and proliferation, as well as cellular response regulation of hypoxia, stretch, stress, and ischemia. Intercellular adhesion molecule 1 (ICAM-1), vascular cell adhesion molecule (VCAM-1), and endothelial leukocyte adhesion molecule-*1* (*ELAM*-*1*) adhesion molecules are upregulated by the transcription factor NF-κB. NF-κB is implicated in several cardiovascular diseases including myocardial ischemia, myocardial reperfusion injury, atherosclerosis, ischemic preconditioning, cardiac hypertrophy vein graft disease, and cardiac failure [61].

Thrombosis results in vessel occlusion and contributes to the pathogenesis of cardiovascular diseases. The tissue factor is a glycoprotein that has an integral role in the triggering of blood coagulation through the extrinsic pathway and acts as a receptor for coagulation factor VII and activated factor VII that could initiate the coagulation cascades; therefore, tissue factor can promote thrombosis in coronary heart disease. Wang et al. used a decoy to bind or “trap” NF-κB and prevent the transcription of the tissue factor gene. In one study, human umbilical cord vein endothelial cells (HUVECs) were transfected with NF-κB transcription factor decoy oligodeoxynucleotides (TFD) using liposomes, and the results showed that the NF-κB decoy competed with the endogenous NF-κB site sequence in the tissue factor promoter for binding to transcription factor NF-κB to block expression of the tissue factor gene [20].

NF-κB regulation may suppress abdominal aortic aneurysm (AAA) progression, and inhibition of NF-κB can protect against AAA development. Akimoto et al. developed an NF- kB decoy to inhibit NF-κB and delivered this decoy to AAA-induced rats via a bioabsorbable sheet. The bioabsorbable sheet delivered the decoy into the target tissues, and treatment resulted in the decoy decreasing the size of the aneurysm, compared with the controls [21].

NF-κB modulates gene transcription involved in myocardial ischemia-reperfusion injury. After extended myocardial preservation blocking NF-κB reduces ischemia-reperfusion injury and improves cardiac function. Sakaguchi et al. transferred NF-κB decoy into rat hearts via a hemagglutinating virus of Japan (HVJ)-liposome vector. NF-κB decoy therapy enhanced the recovery of left ventricular function by lowering serum creatine phosphokinase, neutrophil infiltration, tissue IL-8, and myocardial water content, compared to the control group [22].

NF-κB activates cytokines, adhesion molecule genes, and inducible NO synthase (iNOS). Inflammation underlies myocarditis pathogenesis; therefore, Yokoseki et al. developed a decoy against the *cis*-element of NF-κB to inhibit the progression of experimental autoimmune myocarditis (EAM) and infused decoy into the rat coronary artery. Their results showed that treatment lowered ratios of myocarditis-affected areas to the ventricular cross-sectional area and decreased expression of ICAM-1, iNOS, IL-2, and TNF in the myocardium [23].

Acute transplant rejection or graft arteriopathy may complicate cardiac transplantation and attenuate survival. NF-κB is responsible for gene transcription of inflammatory or proliferative mediators. Suzuki et al. used an HVJ-AVE-liposome to inject an intraluminal NF-κB decoy into cardiac allografts of a murine model. They showed acute rejection of allografts from the major mismatch group, while the NF-κB decoy enhanced graft survival. Migration of cells, the thickness of intima, and inflammatory factors were increased in control allografts of the minor mismatch group, which were decreased by the NF-κB decoy. Treatment by decoy reduced cell infiltration, as well as ICAM-1, VCAM-1, NF-κB, and major histocompatibility complex (MHC) class I and II in allograft myocardium [24].

Sawa et al. used an NF-κB decoy for the attenuation of ischemia-reperfusion injury in the myocardium. NF-κB decoy was transferred to the rat hearts by coronary infusion of HVJ-liposome at the time of the cardioplegic arrest. The percentages of recovery of left ventricular pressure and coronary flow were higher in the NF-κB decoy group when exposed to ischemia and reperfusion. Neutrophil adherence to endothelial cells and levels of interleukin-8 were lower in the treatment group [25].

Morishita evaluated the effect of an NF-κB decoy as an effective therapy for myocardial infarction in rats. The results showed that in vivo transfer of NF-κB decoy before and after infarction, before occlusion of the coronary artery, or immediately after reperfusion decreased the myocardial infarction area. Furthermore, decoy therapy reduced the expression of cytokines (IL-6 and IL-8) and adhesion molecules (VCAM and ICAM) [26].

Nakashima et al. designed a novel chimeric decoy strategy to inhibit NF-B and Ets simultaneously for the treatment of AAA. Chimeric decoy inhibited AAA progression, decreased aneurysmal dilation, reduced MMP expression, and inhibited macrophage migration. Furthermore, it inhibited elastin fiber destruction in the aorta. Importantly, the chimeric decoy ODN inhibited aneurysmal dilatation more than the NF-B decoy [27].

Kalinowski et al. transfected an NF-κB decoy into hypercholesterolemic rabbits in order to prevent restenosis following balloon angioplasty. The NF-κB decoy inhibited proliferation of VSMC in vitro; however, the reduction of the neointimal area by the NF-κB decoy did not differ, compared to the control group [28].

By use of genetically engineered adenoviral (Ad) vectors, bone marrow-derived dendritic cells (DCs) expressed immunosuppressive molecules for promoting T cell unresponsiveness. NF-κB decoy treatment can suppress the maturation of DC through the NF-κB pathway. Bonham et al. used the combination of an NF-κB decoy and rAd vectors encoding CTLA4-Ig (Ad CTLA4-Ig) to produce stably immature murine myeloid DCs that secreted the co-stimulation blocking agent. The capacity of these DCs for stimulation was disrupted resulting in enhanced apoptosis of T cells. Moreover, administration of these DCs before transplantation prolonged survival of MHC-mismatched vascularized heart allografts [29].

Zhang et al. examined whether an NF-κB decoy could further augment the inhibition of cellular proliferation by radiation of VSMCs in vitro. The irradiation promotes activation or nuclear translocation of NF-κB in VSMCs and transfection of the NF-κB decoy inhibited the radiation-induced NF-κB activation in VSMCs. Transcription and translocation of NF-κB downstream molecules including ICAM, iNOS, and TNF-α were reduced. Inhibition of NF-κB by decoy increased apoptosis and reduced proliferation and survival in the irradiated VSMCs [30].

Miyake et al. investigated the effect of a chimeric NF-κB and Ets decoy on aortic dilatation in a rabbit AAA model. Results showed that the chimeric decoy inhibited the aortic dilatation progression that was confirmed histologically. The chimeric decoy also decreased the activities of MMP-2 and MMP-9 and inhibited elastin proteolysis. Furthermore, suppressing VCAM-1 and MCP-1 gene expression by the chimeric decoy inhibited macrophage infiltration in the media and adventitia [31].

DC can modulate immune responses by altering the transcription of NF-κB-regulated genes of surface costimulatory molecules (CD80, CD40, CD86). Tolerance to DC has been related to impaired NF-κB-dependent transcription of costimulatory genes and NF-κB translocation to the nucleus [32]. Giannoukakis et al. demonstrated that an NF-κB decoy was incorporated by bone-marrow-derived DC and the decoy inhibited LPS-induced nitric oxide production, which is a maturation marker of DCs. Treatment by this decoy repressed the cell-surface expression of CDs without affecting MHCs expression. Additionally, decoy treatment inhibited the production of the Th1- type cytokine. Importantly, infusion of the NF-κB decoy DC into allogeneic recipients before heart transplantation prolonged allograft survival in the absence of immunosuppression [32].

Myocardial reperfusion injury is the result of inflammation following ischemia that allows subacute polymorphonuclear leukocytes (PMNs) adhesion, for example, via NF-κB activation. Kupatt et al. studied the effect of targeted NF-κB decoy treatment in the area at risk (AAR) on the size of infarct and regional myocardial function. Liposomes containing NF-κB ODN were retroinfused into the anterior interventricular vein in pigs. NF-κB decoy retroinfusion decreased infarct size, while functional reserve of the AAR tended to improve; therefore, the NF-κB decoy provided postischemic cardioprotection in pig hearts [33].

NF-κB mediates the vascular response to injury, though its role in the mechanism of in-stent restenosis has not been clarified [34]. Ohtani et al. tested the blockade of NF-κB by stent-based delivery of an NF-κB decoy for the reduction of in-stent neointimal formation in rabbits with hypercholesterolemia. Results showed that infiltration of monocytes and expression of MCP-1 were reduced by the NF-κB decoy, while CD14 activation on circulating leukocytes was suppressed by the decoy. Importantly, the neointimal formation was attenuated by the NF-κB decoy. Moreover, transfection of the NF-κB decoy inhibited the proliferation of human coronary artery SMCs in vitro [34].

Miyake et al. developed a modified chimeric decoy against NF-κB and Ets as a novel therapeutic approach for AAA. The decoy had a ribbon-shaped circular structure that had not been chemically modified to maximize its resistance against endonuclease for systemic administration. Ribbon-type decoy ODN (R-ODNs) was administrated intraperitoneally in an elastase-induced rat AAA model. Interestingly, R-ODN inhibited aortic dilatation, although a conventional phosphorothioate chimeric decoy ODN (PS-ODN) failed to prevent the aneurysmal development. Moreover, R-ODN inhibited the activation of MMP-12 and MMP-9 in the aneurysm wall and suppressed the secretion of cathepsin K and B from macrophages, but it did not inhibit the recruitment of macrophages. Chimeric R-ODN also prevented aortic dilatation, while aneurysm development was not prevented by conventional phosphorothioate decoy ODN treatment [39].

Kimura et al. studied the activity of NF-κB in a rat model of PAH. They showed that the NP-mediated NF-κB decoy delivery into lungs prevented monocrotaline-induced NF-κB activation. Blockade of NF-κB by NP-mediated delivery of the NF-κB decoy attenuated inflammation and proliferation, attenuating the development of PAH and pulmonary arterial remodeling induced by monocrotaline [59].

Aoik et al. examine the regressive effect of a chimeric decoy, which simultaneously inhibited NF-κB and Ets-1, on IA development in the rat model [38]. They had previously investigated the expression and role of Ets-1 in CA development using decoy ODN. Treatment with Ets decoy oligodeoxynucleotides resulted in the prevention of CA enlargement, upregulation of MCP-1 expression, and increase in macrophage accumulation in CA walls [41]. Chimeric decoy ODNs decreased intracranial aneurysm (IA) size and thickened IA walls of preexisting IAs induced in the rat model, although the treatment with NF-κB decoy ODNs failed to regress preexisting IAs. Chimeric decoy ODN-treated rats exhibited decreased expression of monocyte chemotactic protein-1 and macrophage infiltration in IA walls [38]. Aoik et al. also showed that treatment with NF-κB decoy restored the reduced expression of procollagen type I, III, and LOX [62]. This group showed that NF-κB decoy prevented CA formation when it was administered at the early stage of aneurysm formation in rats. Macrophage infiltration and expression of downstream genes were dramatically inhibited by NF-κB decoy oligodeoxynucleotide [35].

Miyake et al. used chimeric decoy containing consensus sequences of both NF-κB and Ets binding sites to treat AAA. Inhibitory effects of chimeric decoy on MMP-1 and -9 expressions were confirmed by ex vivo experiments using a human aorta organ culture. Importantly, treatment with chimeric decoy ODNs significantly decreased the size of AAA [37].

Shiraya et al. showed that transfection of a chimeric decoy inhibited aortic dilatation, both in normotensive and hypertensive rats. Destruction of elastic fibers was also inhibited by transfection of the chimeric decoy in both hypertensive rats and normotensive rats. The expression of MMP-2, -3, -9, and -12, as well as the intercellular adhesion molecule, was significantly attenuated by the chimeric decoy ODN, accompanied by inhibition of the migration of macrophages [36].

### 4.2. Activator Protein-1 (AP-1)

AP-1 is a transcription factor involved in the transcriptional regulation of genes implicated in cell proliferation and extracellular matrix production in response to inflammatory cytokines and oxidative stress [42].

Transplant vasculopathy (TV) resulting from cardiac transplantation results in obstructive lesions in vessels. Activation of AP-1 increases the migration and proliferation of SMC. Remes et al. examined AAV-mediated delivery of an RNA hairpin AP-1 decoy oligonucleotide for the treatment of TV in a mouse aortic allograft model of TV. AP-1 decoy oligonucleotides were expressed in the cells of graft tissue. Ex-plantation after 30 days and evaluation showed that AP-1 decoy oligonucleotide therapy reduced the intima-to-media ratio in the grafts by 41.5 percent. Additionally, in treated grafts, expression of cytokines and adhesion molecules were greatly decreased, as were MMP-9-positive cells, SMCs, and inflammatory cell infiltration [42].

Infusion of decoy oligodeoxynucleotides against the activator protein-1 (AP-1) binding site (dec-ODN) prevents neointimal proliferation and thickening [43]. Lin et al. delivered a decoy against AP-1 in-stent and evaluated the inhibitory effects on restenosis. Decoy drug-eluting stents (DESs) were implanted and were compared to normally implanted stents in the abdominal aorta of rabbits. The decoy transmitted in-stent inhibited TGF-1 and CTGF expression, as well as preventing neointimal thickening and restenosis eight weeks after stent implantation. Re-endothelialization was not affected by the decoy [43].

Endothelin-1 synthesis in vessel walls is a powerful co-mitogen for VSMCs stimulating restenosis following percutaneous transluminal coronary angioplasty (PTCA). Deformation-induced expression of prepro-endothelin-1 is governed by AP-1. Buchwald et al. developed a decoy against AP-1 that was given into the coronary arteries of hypercholesterolemic minipigs during PTCA. Decoy treatment decreased neointimal development in coronary arteries, which was correlated with a reduction in AP-1 nuclear translocation and endothelin-1synthesis in the vessel wall [44].

Arif et al. reduced aortic elastolysis through decreased MMP expression with decoy ODNs neutralizing AP-1. Exposure to AP-1 neutralizing dODNs resulted in a significant reduction of basal and interleukin-1β-stimulated MMP expression and activity in mAoSMCs. Moreover, increased migration and formation of superoxide radical anions were substantially decreased in mAoSMCs by AP-1 dODN treatment [45].

### 4.3. Signal Transducer and Activator of Transcription-1 (STAT-1)

STATs proteins are downstream of the Janus kinases (JAKs) signaling pathway. Dysregulation of JAK-STAT signaling has been shown to be related to cardiovascular diseases [63].

Acute myocardial rejection is notable by enhanced leukocyte migration into the graft myocardial tissue. AP-1 and STAT-1 are transcription factors that regulate the expression of vascular adhesion molecules and therefore very important in this process. Hölschermann et al. investigated the effect of a decoy ODN that targeted transcription factors AP-1 and STAT-1 on acute cardiac allograft rejection in a rat transplantation model. They transplanted Wistar–Furth cardiac allografts into Lewis rats following perfusion with AP-1 or STAT-1 dODN solution (5 μmol/L). Treatment with AP-1 and STAT-1 decoys extended cardiac allograft survival by approximately 40%. Immunohistochemical results showed a significant reduction of infiltrating leukocytes, specifically T cells. Furthermore, the expression of ICAM-1 and VCAM-1 in the endothelium was found to be noticeably reduced by decoys. Therefore, both STAT-1 and AP-1 decoys suppressed graft endothelial adhesion molecule expression, diminished graft infiltration, and delayed acute rejection substantially [46].

Stojanovic et al. assessed the STAT-1 decoy in heterotopic mouse heart transplantation without immunosuppression. Mouse heart allografts vessels were pre-treated with STAT-1 decoy. A single treatment with STAT-1 decoy decreased both rejection scores by 85% and 70% in dODN-treated allografts. Furthermore, graft myocardium infiltration by monocyte and T cells was reduced. In addition, STAT-1 decoy impaired pro-inflammatory gene expression greater than 80% 24 h post-transplantation, but the signal was lost 9 days after transplantation [47].

### 4.4. The E2 Factor (E2F)

Abnormal VSMC proliferation and migration has a key role in the pathogenesis of cardiovascular disease. The organized activation of cell cycle regulatory genes is necessary for this process. E2F is a transcription factor that has a key role in cell proliferation regulation, which induces coordinated transactivation of genes involved in cell cycle regulation including c-myb, c myc, cdc2, thymidine kinase, and PCNA.

Cardiac allograft arteriosclerosis is a process involving proliferative SMCs. Transcription factors such as NF-κB and E2F are critical in the transcription of key genes involved in cell proliferation and inflammation. E2F regulates the SMC proliferation of cell-cycle regulatory genes, while NF-κB coordinates the transcription of multiple inflammatory genes [48]. Kawauchi et al. transfected an E2F decoy into monkey heart allografts and performed heterotopic primate cardiac transplantation. After 28 days, transplanted hearts were analyzed and showed that decoy treatment suppressed NF-κB expression in the mildly thickened arterial intima [48]. Suzuki et al. also tested the effect of an E2F decoy for suppressing E-selectin expression in allograft arteries in mice and showed that the E2F decoy limited E-selectin expression on endothelial cells in the thickened intima of the allografts [49].

Kawauchi et al. evaluated the effect of an E2F decoy and antisense cyclin-dependent kinase (cdk2) kinase ODN by ex vivo single intraluminal delivery into cardiac allografts of mice and Japanese monkeys through the HVJ, an artificial viral envelope–liposome method. Results showed that in mice the E2F decoy suppressed neointimal formation and prevented the expression of cell-cycle regulatory genes including PCNA, c-myb, cdk2, and cdc2 until 8 weeks, while antisense cdk2 kinase ODN had minor consequences. In primate models, E2F decoy markedly suppressed neointimal thickening and prevented cell-cycle regulatory genes; however, intimal thickening developed in control groups, suggesting that the E2F decoy could prevent graft arteriopathy without any systemic side effect [50].

### 4.5. Other Targets

Cardiac sarcoplasmic reticulum Ca^2+^-ATPase (SERCA2a) has a vital role in the control of Ca^2+^ in cardiomyocytes. SERCA2a can be inhibited by an endogenous inhibitor named Phospholamban (PLB) that is activated through dephosphorylation by protein phosphatase 1 (PP1). Thus, inhibition of PLB dephosphorylation would be an effective strategy for increasing SERCA2a activity in failing hearts [51]. Oh et al. developed a peptide decoy named ΨPLB-SE and ΨPLB-TE that mimic phosphorylated PLB and serving as a peptide decoy for PP1 leading to the competitive inhibition of the PP1-mediated dephosphorylation of PLB. They found that rat cardiomyocytes treatment with ΨPLB-SE or ΨPLB-TE increased PLB phosphorylation. Moreover, the perfusion of isolated rat hearts with ΨPLB-SE or ΨPLB-TE increased left ventricular pressure ex vivo that had been affected by ischemia. Accordingly, these decoys could be effective for the restoration of SERCA2a activity in failing hearts [51].

MicroRNAs are potential therapeutic targets that normalize gene expression in various diseases. Jeong et al. demonstrated that miR-25 has a key role in the regulation of SERCA2a expression. In a murine heart failure model, they showed that antagomirs against miR-25 increased cardiac contractility and function through SERCA2a restoration. They also used tough decoy (TuD) as an effective long-term suppressor of miR-25 due to their endonuclease resistance, and a highly effective method for microRNA inhibition, which is more resistant to degradation than antagomirs. They developed a miR-25 TuD inhibitor and subcloned it into a cardiotropic AAV9 vector. The TuD selectively inhibited the miR-25 in cardiomyoblast culture. Furthermore, in vivo analysis showed that AAV9-TuD that delivered to the pressure-overload heart failure mouse model selectively reduced miR-25 expression, increased protein levels of SERCA2a, and improved cardiac dysfunction and fibrosis [52].

The neuregulin-1-ErbB pathway is important for maintaining cardiac function. miR-146a was demonstrated to be upregulated by Dox in neonatal rat cardiac myocytes. Horie et al. showed that miR-146a targets the ErbB4. To study the loss of miR-146a function, they used decoys with tandem complementary sequences for miR-146a in the 3′UTR of a luciferase gene. When miR-146a “decoy” genes were introduced into cardiomyocytes, ErbB4 expression was upregulated and Dox-induced cell death was reduced [56].

Overexpression of miR-133 in vitro inhibited cardiac hypertrophy. Dong et al. suppressed miR-133 by “decoy” sequences to induced hypertrophy. Therefore, changes of miR-133 were considered as the requisites for determining cardiac hypertrophy. Results showed that miR-133 expression was downregulated, and calcineurin activity was enhanced in both in vivo and in vitro cardiac hypertrophy models [57].

Restenosis limits percutaneous transluminal coronary angioplasty that is well established for the treatment of advanced coronary artery disease. Medial smooth muscle cells (SMCs) proliferation and migration are the key causes of this process. CCAAT/enhancer-binding protein (C/EBP) binds to several genes that encode acute-phase proteins. Hence, C/EBP is a promising target for preventing restenosis following balloon angioplasty. Kelkenberg et al. used a decoy ODN to neutralize C/EBP. This decoy was administered for 30 min to the site of injury, which was generated using a balloon, and cholesterol-mediated chronic inflammation in a rabbit model of restenosis. Results showed that after 2 days, C/EBP activity in treatment segments was almost absent. After 28 days, decoy reduced neointimal formation and intravascular inflammation (up to 50%). On day 3, de novo endothelin-1 synthesis and the amount of proliferating cell nuclear antigen-positive SMCs were both significantly reduced in the vessel wall [53].

Inflammatory cell recruitment in the ischemic heart depends greatly on CC chemokines and their receptors [54]. Cochain et al. investigated whether the chemokine decoy receptor D6 that binds to and scavenges inflammatory CC chemokines may help to reduce inflammation and avoid adverse cardiac remodeling following infarction. D6 was observed to be expressed in both murine and human infarcted myocardium. D6-/-mice with myocardial infarction had higher levels of chemokine ligand 2 and chemokine ligand 3 in the ischemic heart. Pathogenic neutrophils and Ly6Chi monocytes infiltration were increased in D6-/-infarcts, which was associated with increased MMP-9 and MMP-2 activities in the ischemic heart. After myocardial infarction, D6-/-mice were more susceptible to cardiac rupture. Further analysis showed that D6/-hearts had characteristics of adverse remodeling including left ventricle dilation with reduced ejection fractions. Additionally, as indicated in bone marrow chimera experiments leukocyte-borne D6 did not play a role in this setting, while the lack of leukocyte-specific chemokine (C-C motif) receptor 2 rescued the adverse phenotype observed in the D6-/-mice model [54].

Apoptosis is involved in dilated cardiomyopathy (DCM) [55]. Schoppet et al. assessed the involvement of the apoptosis-inducing cytokine TRAIL (TNF-related apoptosis-inducing ligand) and its decoy receptor osteoprotegerin (OPG) in the pathogenesis of DCM. The expression of TRAIL and OPG were assessed in nonischemic DCM patients without coronary artery disease after coronary angiography and endomyocardial biopsy (EMB). TRAIL and OPG protein were identified in EMB of DCM patients but not in controls. Real-time PCR indicated the upregulation of TRAIL mRNA in peripheral blood leukocytes (PBL)and OPG mRNA in endomyocardial specimens. Therefore, antiapoptotic OPG in the myocardium of DCM patients overexpressed to limit systemic activation of TRAIL as a compensatory mechanism in patients with congestive heart failure [55]. Potentially, OPG as decoy-based treatment may have utility as treatment for DCM patients.

Activating autoantibodies to the angiotensin II type 1 receptor (AT1R) have been implicated in hypertensive disorders. Li et al. studied the therapeutic potential of a newly designed retro-inverso d-amino acid (RID) decoy peptide that specifically targets AT1R-AAbs. This decoy prevents the autoantibody from binding to the cell membrane receptor and keeping it from activation. They showed that this would lead to clearance of the decoy peptide–autoantibody complex and potentially lead to the body developing tolerance to this epitope and suppression of autoantibody production [58].

Elevated thrombospondin 1 (TSP1) is a factor in the pathogenesis of cardiovascular conditions, including ischemia-reperfusion injury (IRI) and pulmonary arterial hypertension (PAH), via cognate receptor CD47. Yao et al. designed a decoy receptor protein to specifically bind TSP1 and neutralize TSP1-impaired vasorelaxation, strongly implicated in IRI and PAH. They showed that this decoy has the great potential to abrogate TSP1-associated cardiovascular complications [60].

Miwa et al. evaluated the role of Ets in a rat AAA model using a decoy. Results showed that the decoy decreased MMPs expression and suppressed aneurismal dilation. Moreover, the destruction of elastin fibers was inhibited in the aorta transfected with Ets decoy ODN, accompanied by a reduction of MMP-1 and -9 expression [40].

## 5. Conclusions

Despite the development of several therapeutic approaches, CVD remains a major global health problem. Recently, decoy technology, including decoy ODN and peptide decoy, has shown great potential as effective therapeutic tools in cardiac disease.

Decoy ODN is a method to suppress gene activation by trapping the transcription factor of target genes, thus inhibiting transcription. The great advantage of decoy ODN is that it can be synthesized easily and can be widely applied to many genes. The efficiency of decoy ODN against critical transcription factors such as AP-1, NF-κB, and E2F shows potential utility in the treatment of several CVDs.

Decoy peptide treatment is an effective tool for neutralizing a specific peptide that has a key role in a pathological condition. Decoy peptides in some fields such as virus infection (for trapping viruses through binding to the surface ligands) have been studied; however, for CVDs only a few decoy peptides such as ΨPLB-SE/ΨPLB-TE decoy, chemokine decoy receptor D6, and decoy receptor OPG have been examined to date and more studies are needed.

The effective delivery and stability of decoys as therapeutic tools in CVD needs further evaluation to determine if they have a place in mainstream therapeutic strategies.

## Figures and Tables

**Table 1:** Therapeutic effects of oligodeoxynucleotides (ODN) and peptide decoy on non-atherosclerotic CVDs.

Decoy Name	Type of CVD	Target Up (↑)/Down (↓)	Concentration/Dose µM mg/kg/day	Decoy Type	Model/Cell Line	Experimental Replications Treatment/Control	Delivery Method	Results	Ref.
NF-κB decoy	Thromboembolism diseases	NF-κB ↓ Tissue factor ↓	100 µmol/L	ODN	HUVEC	-	Liposomes	Blocked expression of tissue factor gene	[20]
NF-κB decoy	Abdominal aortic aneurysm	NF-κB ↓		ODN	SD rats that were induced with abdominal aortic aneurysm	5/5	Bioabsorbable sheet	Reduced the aneurysm size	[21]
NF-κB decoy	Myocardial ischemia-reperfusion injury	NF-κB ↓	10 µmol/L	ODN	Rats	6/6	HVJ liposomes	Reduced ischemia-reperfusion injury after prolonged heart preservation	[22]
NF-κB decoy	Autoimmune myocarditis	NF-κB ↓	15 μmol/L	ODN	Rats	8/6	HVJ-AVE-Liposomes	Reduces the severity of EAM	[23]
NF-κB decoy	Cardiac allograft rejection	NF-κB ↓	-	ODN	Mice	6/6	HVJ-AVE-Liposomes	Reduced myocardial cell infiltration and arterial intimal thickening	[24]
NF-κB decoy	Myocardial ischemia-reperfusion injury	NF-κB ↓	-	ODN	Rats	6/6	HVJ-liposome	The percentages of recovery of left ventricular developed pressure and coronary flow were higher	[25]
NF-κB decoy	Myocardial infarction	NF-κB ↓	1–10 µM	ODN	Rats	5/5	HVJ-liposome	Decreased the myocardial infarction area and reduced the expression of adhesion molecules and cytokines including IL-8 and IL-6	[26]
Chimeric decoy ODN	Abdominal aortic aneurysm	NF-B ↓ Ets ↓	100 nmol/cm^2^	ODN	Rat	6/6	-Cellulose and polyethylene glycol	Inhibited AAA progression, reduced MMP expression, and inhibited macrophage migration The effect of Chimeric decoy was greater than NF-B decoy	[27]
NF-κB decoy		NF-B ↓							
NF-B decoy	Restenosis	NF-B ↓	100 ng	ODN	hypercholesterolemic rabbits	14/8	Liposomal carrier (TfX50)	Did not reduce neointimal hyperplasia	[28]
NF-B decoy	Cardiac allograft rejection	NF-B ↓	10 µM	ODN	mice	3/3	-	Suppressed DC maturation	[29]
NF-B decoy	Restenosis	NF-B ↓	2 µmol/l decoy	ODN	VSMCs	-	Lipofectamine	Increased apoptosis and reduced proliferation and survival in the irradiated VSMCs	[30]
Chimeric decoy NFkB decoy Ets-1 decoy	Abdominal aortic aneurysms	NF-B ↓ Ets ↓	100 nmol decoy/cm^2^	ODN	Elastase-induced rabbit AAA model	9/9	delivery sheet (hydroxypropyl cellulose and PEG)	Prevented aortic dilatation progression and decreased the activities of MMP-9 and MMP-2	[31]
NF-κB decoy	Cardiac allograft rejection	NF-B ↓	10 µM	ODN	Mice, Bone marrow-derived DC	10/6	-	Repressed the cell-surface expression of costimulatory molecules and prolonged allograft survival	[32]
NF-κB decoy	Myocardial ischemia-reperfusion injury	NF-B ↓	75 nmol per heart	ODN	Pig	-	Liposome	Decreased infarct size	[33]
NF-κB decoy	Restenosis	NF-B ↓	500 to 600 µg per stent	ODN	Hypercholesterolemic Rabbit, SMCs	3/3	Polyurethane	Attenuated neointimal formation and suppressed proliferation of human coronary artery SMCs	[34]
NF-κB decoy	Aneurysms	NF-κB ↓	-	ODN	Rats	10/7	-	Prevented CA formation and macrophage infiltration	[35]
NF-κB decoy	Aneurysms	NF-κB ↓	-	ODN	Rats	15/15	-	Inhibited aortic dilatation and migration of macrophages	[36]
Chimeric decoy	Aneurysms	NF-κB ↓ Ets-1 ↓	-	ODN	Rabbits	24/24	-	Decreased the size of AAA	[37]
Chimeric decoy	Aneurysms	NF-κB ↓ Ets-1 ↓	-	ODN	Rats	9/10	-	Decreased IA size and thickened IA walls	[38]
R-ODN	Abdominal aorticaneurysm	NF-B↓Ets-1 ↓	50 nmol/week	ODN	AAA model rat	9/9	-	Suppressed the activation of MMP-9 and MMP-12R ODNs but not PS-ODN prevented aneurysm formation	[39]
PS-ODN									
Ets-1 decoy	Aneurysms	Ets-1 ↓	-	ODN	Rats	8/86/6	Delivery sheet	Decreased MMPs expression and suppressed aneurismal dilation	[40]
Ets-1 decoy	Aneurysms	Ets-1 ↓	-	ODN	Rats	8/8	-	Prevented CA enlargement and upregulation of MCP-1 expression	[41]
AP-1 decoy	Cardiac allograft transplant vasculopathy	AP-1 ↓	-	Decoy oligonucleotide	Mouse transplant vasculopathy model	9/9	AAV	Reduced intima-to-media ratio, reduced the expression of cytokines, adhesion molecules and decreased the numbers of proliferative SMCs, MMP-9-positive cells, and inflammatory cell infiltration	[42]
AP-1 decoy	Restenosis	AP-1 ↓	20 ug/mL	ODN	Rabbit VSMCs	10/10	-	Inhibited the expression of TGF-1 and CTGF and inhibited neointimal thickening and restenosis	[43]
AP-1 decoy	Restenosis	AP-1 ↓	20 nmol	ODN	Hypercholesterolemic minipigs	3–12/10	-	Reduced neointimal formation	[44]
AP-1 decoy	Aneurysms	AP-1 ↓	10 μmol/L	ODN	Mice	7/7	-	Reduction of basal and interleukin-1β-stimulated MMP expression and activity in mAoSMCs	[45]
AP-1 decoy	Cardiac allograft rejection	AP-1 ↓	5 μmol/l	ODN	Ex vivo Rat	5/5	-	Suppressed graft, reduced graft infiltration and delayed acute rejection	[46]
STAT-1 decoy		STAT-1 ↓							
STAT-1 decoy	Acute rejection of heart transplants	STAT-1↓	10 µM	ODN	Mice	3/3	-	Decreased rejection scores and reduced infiltration of monocyte and T cells	[47]
E2F decoy	Cardiac allograft arteriosclerosis	E2F ↓ NF-κB ↓	-	ODN	Monkeys	5/4	HVJ-liposome	Blocked the NF-κB expression in the mildly thickened arterial intima	[48]
E2F decoy	Cardiac allograft arteriopathy cardiac transplantation	E2F ↓ E-selectin ↓	-	ODN	Mice	-	HVJ-liposome	Limited E-selectin expression	[49]
E2F decoy	Neointimal formation cardiac allograft arteriopathy	E2F ↓	-	ODN	mice and Japanese monkeys	5/5	HVJ liposome	Suppressed neointimal formation, prevented expression of cell-cycle regulatory genes and reduced Cardiac allograft arteriopathy	[50]
ΨPLB-SEΨPLB-TE	Heart failure	protein phosphatase 1 ↓	1 μM for 4 h	Peptide	Cardiomyocytes	-	cell-permeable peptide (TAT)	Increased PLB phosphorylation, restored activity of SERCA2a and improved recovery after ischemia/reperfusion in the heart.	[51]
Tough decoy (TuD)	Heart failure	miR-25 ↓	300 mg	ODN	SERCA2 KO mice/cardiomyoblast	5/5	AAV9 vector	Increased protein levels of SERCA2a and improved cardiac dysfunction and fibrosis	[52]
C/EBP decoy	Restenosis	C/EBP ↓ Endothelin-1 ↓	20 μmol/l	ODN	Hypercholesterolemic Rabbit	7/7	-	Reduced neointimal formation and intravascular inflammation	[53]
Chemokine decoy receptor D6	Adverse ventricular Remodeling following myocardial infarction	inflammatory CC-chemokines↓	-	Peptide	Myocardial infarcted D6-/-murine model	6/6	-	Limited CC-chemokine-dependent pathogenic inflammation that is needed for adequate cardiac remodeling following myocardial infarction	[54]
Decoy receptor osteoprotegerin (OPG)	Dilated cardiomyopathy	TRAIL↓	-	Peptide	Human	24/22	-	Limited systemic activation of TRAIL	[55]
miR-146a	Chemotherapy-induced cardiotoxicity	miR-146a ↓ ErbB4↑	-	ODN	Mice	-	-	Up-regulated ErbB4 expression and reduced Dox-induced cell death	[56]
miR-133 decoy	Cardiac hypertrophy	miR-133 ↓	-	ODN	Primary cardiomyocytes, Rats	5/5	Lipofectamine 2000	Downregulated *miR-133* expression, and enhanced calcineurin activity	[57]
RID decoy	Hypertensive disorders	AT1R-AAbs ↓	1 mg/kg	Peptide	Rabbits	6/6	-	Led to the body developing tolerance to this epitope and suppression of autoantibody production	[58]
NF-κB decoy	Hypertensive disorders	NF-κB ↓	50 µg	ODN	Rat model of PAH	33/33	PEG-PLGA	Attenuated the development of PAH	[59]
TSP1 decoy	Hypertensive disorders	TSP1 ↓	-	Peptide	Mice	5/7	-	Abrogated TSP1-associated cardiovascular complications	[60]

Abbreviations used in this table: AAA: abdominal aortic aneurysm; AP-1: activator protein-1; DC: dendritic cells; HVJ liposomes: hemagglutinating virus of Japan liposomes; IL: interleukin; MCP-1: monocyte chemoattractant protein-1; MMP: matrix metalloproteinases; NF-κB: nuclear factor-kappa B; ODN: oligodeoxynucleotides; PLB: phospholamban; VSMCs: vascular smooth muscle cells; SD: Sprague–Dawley; SMCs: smooth muscle cells.

## Data Availability

Not applicable.
